# Translation of gastric disease progression at gene level expression

**DOI:** 10.7150/jca.29038

**Published:** 2020-01-01

**Authors:** Stephanie Euridice Morales-Guerrero, Claudia Ivette Rivas-Ortiz, Sergio Ponce de León-Rosales, Armando Gamboa-Domínguez, Claudia Rangel-Escareño, Luis Federico Uscanga-Domínguez, Germán Rubén Aguilar-Gutiérrez, David Kershenobich-Stalnikowitz, Gonzalo Castillo-Rojas, Yolanda López-Vidal

**Affiliations:** 1 Programa de Inmunología Molecular Microbiana, Departamento de Microbiología y Parasitología, Facultad de Medicina, Universidad Nacional Autónoma de México (UNAM), Ciudad de México, México; 2 Dirección de Enseñanza, Departamentos de Gastroenterología, Patología y Dirección General. Instituto Nacional de Ciencias Médicas y Nutrición Salvador Zubirán (INCMNSZ), Ciudad de México, México; 3 Departamento de Genómica Computacional, Instituto Nacional de Medicina Genómica (INMEGEN), Ciudad de México, México; 4 Centro de Investigación Sobre Enfermedades Infecciosas (CISEI), Instituto Nacional de Salud Pública (INSP), Cuernavaca, Morelos, México

**Keywords:** *Helicobacter pylori*, microarray, gene expression, chronic gastritis, follicular gastritis, and intestinal metaplasia.

## Abstract

*Helicobacter pylori* is associated with the development of several lesions in the human stomach. This chronic infection produces gastritis, which can progress to intestinal metaplasia and gastric cancer. To date, there is very little information regarding gene-expression in the different phases of progression caused by chronic *H. pylori* infection. In this study, we performed a genome-wide gene-expression analysis in gastric biopsies of patients chronically infected with *H. pylori,* using the potential of high-throughput technologies that have not been fully exploited in this area. Here we illustrate the potential correlation of *H. pylori* infection with the gene expression changes in follicular gastritis, chronic gastritis and intestinal metaplasia. We also suggest its potential as biomarkers of each condition. An exploratory set of 21 biopsies from patients with follicular gastritis, chronic gastritis, and intestinal metaplasia were analyzed by gene-expression microarrays in order to identify the biological processes altered in each lesion. The microarray data was corroborated by real-time PCR, while 79 Formalin-Fixed Paraffin-Embeded samples were analyzed by immunohistochemistry. Follicular gastritis exhibited significant enrichment in genes associated with glutamate signaling, while chronic gastritis showed a down-regulation in metallothionein 1 and 2 and in oxidative phosphorylation-related genes, which could be associated with the chronic infecton of *H. pylori*. Intestinal metaplasia exhibited an over-expression of gastrointestinal stem cell markers, such as *LGR5* and *PROM1*, as well as messenger RNA and nucleic acid metabolism-related genes. The gene-expression patterns found in this study provide new comparative information about chronic gastritis, follicular gastritis and intestinal metaplasia that may play an important role in the development of gastric cancer.

## Introduction

Gastric adenocarcinoma is an important health problem worldwide. Although there has been a decrease in the incidence of the disease, gastric adenocarcinoma continues to represent the third cause of cancer-related deaths 1. Two variants of gastric adenocarcinoma have been described: diffuse and intestinal 2. The latter has been proposed to be the result of several histological changes associated with *H. pylori* infection, a bacterium described as a Type I Carcinogen by the International Agency for Research on Cancer (IARC) 3.

The first response of the stomach to persistent *H. pylori* infection is chronic gastritis. Since the stomach is unable to clear the infection, it produces a chronic inflammatory environment, defined as chronic gastritis, which is an initial histological change in the development of gastric atrophy and which has been controversially suggested to be reversible 4-9. This gastric atrophy may eventually progress to intestinal metaplasia or gastric adenocarcinoma 4, 5, 6. The follicular gastritis is characterized by the presence of a large number of lymphoid follicles and mononuclear cell infiltration, whilst intestinal metaplasia can lead to the development of adenocarcinoma. The progression from chronic gastritis to gastric adenocarcinoma is a well-accepted model of gastric carcinogenesis, first described by Correa in 1992 6.

The biological processes involved in progression from chronic gastritis to gastric adenocarcinoma remain unclear. Gene-expression analyses of gastric adenocarcinoma have mainly focused on describing the differences between gastric tumors and normal tissue and/or measuring gene-expression between adults and infants 10-22. Despite the knowledge gained from these studies, only a few efforts have been made to determine gene-expression in intestinal metaplasia and chronic gastritis 14, 23-29.

The recent advances in high-throughput technologies and the available bioinformatic tools have made possible to evaluate changes in the expression of the whole genome instead of focusing only on a limited number of genes. Furthermore, they provide a better view of the biological processes involved in a particular disease and identify potential biomarkers 30. This can greatly contribute to understanding the molecular pathogenesis of *H. pylori* and its possible implications in the development of gastric cancer when comparing gene expression of healthy tissue with the gastric lesions that precede gastric adenocarcinoma.

The early diagnosis of gastric adenocarcinoma is an important resource in the improvement of the treatment and survival of patients. Accordingly, the gene expression analyses can help to develop not only early diagnostic tools, but also new early treatments of gastric adenocarcinoma 31.

In this study, we performed a genome-wide gene-expression analysis by microarrays and used new bioinformatic tools in follicular gastritis, chronic gastritis, and intestinal metaplasia in order to identify the altered molecular mechanism and potential biomarkers of each lesion through the identification of characteristic gene-expression profiles.

## Materials and Methods

### Ethics statement

This study was approved by the Investigation and Ethics Committee of the School of Medicine of the UNAM, National Institute of Medical Sciences and Nutrition Salvador Zubirán (INCMNZS), General Hospital of Mexico Dr. Eduardo Liceaga (HGM), and Medical Center ABC (Registry numbers: 019-2009, 209, DIC/10/107/05/119, and ABC-11-16, respectively). All participants gave their written informed consent prior to sample collection.

To reach the aim of the study we collected two sets of samples. The first one, the exploratory set, consisted of gastric biopsies of patients with follicular gastritis, chronic gastritis and intestinal metaplasia, obtained by endoscopy and submitted to microarray analysis. The second set of samples, corresponding to the validation set, consisted of formalin-fixed paraffin-embedded (FFPE) tissues obtained from the sample bank of the pathology department of the INCMNZS. The samples corresponding to follicular gastritis, chronic gastritis, and intestinal metaplasia, as well as stomachs without lesions were analyzed by immunohistochemistry in order to corroborate the results from the exploratory set.

### Exploratory set

The exploratory set of gastric biopsies was collected from patients with follicular gastritis, chronic gastritis and intestinal metaplasia. After their informed consent, subjects with gastric complaints who were programmed for an exploratory endoscopy to determine the source of their symptoms were recruited. After the endoscopic procedure, samples taken for diagnosis were submitted to the pathology department and an expert pathologist performed the histological examination according to the Sydney classification 32. With these pathology results, the patients with a diagnosis of follicular gastritis, chronic gastritis, and intestinal metaplasia were selected. Subjects with a diagnosis that differed from our interest, such as lymphoma or peptic ulcer, were discarded. Besides the routine samples taken, biopsies from the gastric lesions were taken and stored in RNAlater (Ambion, USA) at -70ºC for nucleic acid preservation until use.

### Validation set

The histological records of the sample bank of the INCMNZS from 10 years ago to date were reviewed to identify FFPE blocks corresponding to tissues with follicular gastritis, chronic gastritis, and intestinal metaplasia. Due to the fact that we had available samples from a broad time range, we were also able to collect samples from healthy gastric tissues. Serial 3-µm slices were taken from each block to perform an immunohistochemical analysis.

### Total RNA extraction

The biopsies selected from the exploratory set were removed from RNAlater and put into lysis solution (RNAqueous Kit; Ambion, USA). The tissues were homogenized with a Tissue Ruptor (Cole Parmer, USA) until they were completely lysed. Total RNA was extracted using the RNAqueous Kit (Ambion, USA) per the manufacturer's instructions. Total RNA was eluted in 20 μL of nuclease-free water. RNA quality (A_260_/A_280_, and A_260/_A_230_ ratios) was measured using a NanoDrop ND-1000 spectrophotometer (NanoDrop Technologies, USA). RNA integrity was assessed through the RNA Integrity Number (RIN), using an Agilent 2100 Bioanalyzer (Agilent Technologies, USA). Total RNA samples were stored frozen at -70ºC until use.

### Microarray assays

For microarray experiments, target complementary DNA (cDNA) from each biopsy was prepared according to the WT Expression Sense Target Kit (Ambion, USA). Briefly, one µg of RNA was converted into first-strand cDNA. Next, a second-strand cDNA synthesis was performed, followed by an *in-vitro* transcription to generate cRNA. The cRNA products were used as templates for a second-cycle cDNA synthesis where dUTP was incorporated into the new strand. Purified sense-strand cDNA (with incorporated dUTP) was fragmented and labeled using the Affymetrix GeneChip WT Terminal Labeling Kit (Affymetrix, USA). The cDNA was fragmented using Uracil-DNA Glycosylase (UDG) and Apurinic/Apyrimidic Endonuclease 1 (APE1). The fragments (40-70 mers) were then labeled by means of a biotin-labeled deoxynucleotide terminal addition reaction using a Terminal deoxynucleotidyl Transferase (TdT). Finally, using the GeneChip Hybridization, Wash and Stain Kit, each fragmented and labeled cDNA sample was hybridized onto Affymetrix Human Gene 1.0 ST arrays (Affymetrix, USA), respectively. After hybridization, the 21 arrays were washed, stained for biotinylated cDNA, and scanned per the manufacturer's recommendations in order to obtain CEL files for each microarray.

### Microarray analysis

Samples were classified into three main groups: 1) samples from patients with Follicular Gastritis (FG); 2) samples from patients with Chronic Gastritis (CG), and 3) samples from patients with Intestinal Metaplasia (IM). All possible pairwise comparisons among the three groups generated three contrasts-of-interest: CG *vs.* FG; IM *vs.* FG, and IM *vs.* CG.

Raw data was analyzed to confirm its normal distribution; afterwards it was background-corrected using Robust Multiarray Average (RMA) 33 and normalized using Quantile Normalization 34. The expression matrix created with this procedure was employed for the enrichment analyses and the selection of differentially expressed genes.

### Enrichment analyses

In order to have a general overview of the biological differences among the groups considered in each comparison, we performed Gene Set Enrichment Analysis (GSEA). This analysis allowed us to evaluate if a defined set of genes showed statistically significant differences between two biological states 35, 36. Gene sets corresponding to hallmark gene sets 37, gene ontology terms 38, and pathways, were obtained from the Molecular Signatures Database (MSigDB). GSEA was carried out using default parameters and is available at the Gene Pattern Public Server (https://genepattern.broadinstitute.org/gp/pages/login.jsf).

### Selection of differentially expressed genes

Differential expression was determined using statistical linear models; contrasts-of-interest were analyzed using the Bioconductor Library Limma 39, 40. Correction for multiple hypotheses was applied utilizing the False Discovery Rate (FDR) 41. Genes were selected based on a |Fold-Change in base 2 log scale (FCh)| ≥ 0.3 and a statistical significance per a B-statistic Log odds > -1. Hierarchical clustering was performed to visualize the gene expression patterns of each comparison. The data is available from the NCBI Gene Expression Omnibus Database (GEO) (http://www.ncbi.nlm.nih.gov/geo/) under Accession Number GSE106656.

### Functional annotation of differentially expressed genes

The differentially expressed genes selected for each comparison were uploaded onto DAVID software (Database for Annotation, Visualization and Integrated Discovery; http://david.abcc.ncifcrf.gov/) 42. Using default parameters, gene ontology terms and KEGG Pathways enriched in gene lists were determined.

### Real-Time PCR

The cDNA from each exploratory set of samples was obtained using the Superscript III Kit per the manufacturer's protocol (Invitrogen, USA). TaqMan Gene expression assays (Applied Biosystems, USA) used for the validation of genes-of-interest included the following: *LRP1* = Low density lipoprotein receptor-related protein 1 (Hs00233856_m1); *SLCO2A1* = Solute Carrier Organic Anion Transporter Family Member 2A1 (Hs00194554_m1); *MUC17* = Mucin 17 (Hs00959753_s1); *NDUFS8* = NADH: Ubiquinone Oxidoreductase Core Subunit S8 (Hs00159597_m1): *MT2A* = Metallothionein 2A (Hs02379661_g1); *PROM1* = Prominin 1 (Hs01009250_m1); *LGR5* = Leucine-rich repeat containing G protein-coupled Receptor 5 (Hs00173664_m1); *OLFM4* = Olfactomedin 4 (Hs00197437_m1); *HDAC7* = Histone Deacetylase 7 (Hs00248789_m1), and *PDK1* = Pyruvate dehydrogenase kinase 1 (Hs01561850_m1). *B2M* = Beta-2-Microglobulin (Hs00984230_m1) was used as a housekeeping gene. Real time-PCR was performed according to TaqMan Gene expression assay conditions in an ABI PRISM 7300 Real-Time PCR instrument (Applied Biosystems, USA). Each gene-of-interest and the housekeeping gene were tested in triplicate. Gene-expression differences were assessed through the ∆∆Ct method.

### Immunohistochemistry of Metallothioneins (MTs)

A descriptive analysis for the metallothioneins in each study group was carried out. A positive signal indicating the expression of MTs within the cells (positive signal either in the cytoplasm or nucleus) was evaluated and the distribution of the signal along the gastric tissue was analyzed. Briefly, the FFPE samples from the validation set were dewaxed and rehydrated. Anti-MT antibody (Clone E9; Dako, USA), diluted at 1:100, was used for MTs detection. The Mouse/Rabbit ImmunoDetector HRP/DAB Detection System (Bio SB, USA) was employed to carry out the Immunohistochemistry (IHC). Tissue sections were briefly counterstained with Hematoxylin and were observed under the Olympus BX41 microscope; images were acquired with Q-Pro software (QImaging Scientific, Canada). Negative control of IHC was done omitting the primary antibody against MTs.

## Results

After the histopathological analysis of the samples collected by endoscopy, an exploratory set consisting of 21 biopsy samples was obtained (Table 1). The samples collected were from follicular gastritis (*n* = 7), chronic gastritis (*n* = 7), and intestinal metaplasia (*n* = 7). For the validation set, after reviewing the pathology records, 79 Formalin-Fixed Paraffin-Embeded tissues were obtained (Table 1), The tissues corresponded to follicular gastritis (*n* = 20), chronic gastritis (*n* = 21), intestinal metaplasia (*n* = 20), and gastric tissues without histological lesions (*n* = 18).

For gene-expression analysis, the 21 biopsies of the exploratory set were submitted to microarray analysis. Considering the three groups of samples, we performed three comparisons of gene expression: Follicular Gastritis *vs.* Chronic Gastritis (FG *vs.* CG), Follicular Gastritis *vs*. Intestinal Metaplasia (FG *vs.* IM), and Chronic Gastritis *vs*. Intestinal Metaplasia (CG *vs.* IM). Volcano plots depicted the proportion of genes that were up-regulated and down-regulated in each comparison (Figure S1).

### Follicular gastritis and chronic gastritis display different gene-expression patterns

Histologically, follicular gastritis can be distinguished due to the presence of lymphoid follicles within the lamina propria. This feature is absent in chronic gastritis, where the inflammatory infiltrate does not form a specialized structure. Gene-expression analyses were performed to determine the molecular differences between these two lesions. The hallmark gene-set tool showed different expression patterns between follicular gastritis and chronic gastritis. Although the results possessed no statistical significance, we could identify trends in gene-expression patterns. In follicular gastritis, there is an enrichment of genes involved in oxidative phosphorylation, along with the metabolism of Reactive Oxygen Species (ROS) and fatty acid metabolism. In contrast, in chronic gastritis, the analysis showed an enrichment of immunological processes (Table 2).

When using ontology terms, GSEA revealed an enrichment of ion transporter activity and glutamate signaling in follicular gastritis, while there was an enrichment of GTPase-related terms in chronic gastritis (Dataset S1). Pathway enrichment analysis with GSEA showed that, in follicular gastritis, enrichment in pathways associated with ion metabolism occurs. On the other hand, chronic gastritis displayed enrichment in immunological- and proliferation-related pathways (Dataset S1). This denotes that even though both conditions involve an inflammatory response, they display a different global expression pattern regarding the inflammatory environment. Between chronic gastritis and follicular gastritis, we found 331 differentially expressed genes (Dataset S6.). Of these, 112 were up-regulated and 219 were down-regulated (Figure S1A). Hierarchical clustering exhibited a specific expression pattern, enabling discernment between the samples corresponding to each group (Figure S2A).

There is a sample corresponding to follicular gastritis (P107) that appears to be clustered with the samples corresponding to chronic gastritis. This attracted our attention. However, the heat map also showed that gene expression is related to the samples corresponding to the group of follicular gastritis because we can observe that the pattern of up- and down-regulation is consistent with this group of samples.

Functional annotation employing ontological terms showed that there was a down-regulation of mitochondrial-related genes (Dataset S2). Pathway analysis revealed that oxidative phosphorylation was affected (Table 3). Specifically, genes associated with complexes I, III, and V of the respiratory chain demonstrated a down-regulation in chronic gastritis in comparison with follicular gastritis. The results suggest that the mitochondrion plays a differential role between chronic and follicular gastritis. Interestingly, in chronic gastritis, there was a down-regulation of genes encoding members of Metallothionein 1 and 2.

### Intestinal Metaplasia displayed an alteration in RNA metabolism and stem cell markers

Intestinal metaplasia is considered a stomach lesion developed as a result of the constant inflammation caused by *H. pylori* 43. We analyzed the gene expression of intestinal metaplasia in comparison with both variants of gastritis in order to understand the molecular differences between the lesions. GSEA employing hallmark-gene sets demonstrated a trend toward the enrichment of immunological-related genes in follicular gastritis. Intestinal metaplasia displayed an enrichment of lipid metabolism- and proliferation-related hallmarks (Table 4).

Gene ontology analysis with GSEA identified an interesting enrichment of mRNA metabolism, transcription and Golgi apparatus-related terms associated with intestinal metaplasia. Follicular gastritis showed an enrichment of terms related to the negative regulation of biological processes, as well as an enrichment of glutamate signaling-related terms (Dataset S3). Analysis of pathways in follicular gastritis revealed the enrichment of pathways related to immunological processes. Intestinal metaplasia showed an enrichment of pathways related to nucleic acid and lipid metabolism (Dataset S3). These results correlate with the histological observations where, in gastritis, there is a marked immune response that is absent in intestinal metaplasia.

The comparison of the gene expression of intestinal metaplasia and follicular gastritis yielded 479 differentially expressed genes (Dataset S6.). Of these, 178 were up-regulated and 301 were down-regulated in intestinal metaplasia in comparison with follicular gastritis (Figure S1B). The hierarchical clustering of 479 genes reveals a gene-expression pattern that distinguishes gastritis from metaplasia samples (Figure S2B).

An analysis on DAVID using ontological terms showed that the nucleotide metabolism was altered in intestinal metaplasia (Dataset S4). These findings correlate with previous GSEA results. Analysis of KEGG pathways showed an enrichment of carbohydrate metabolism (Table 3) in intestinal metaplasia in comparison with follicular gastritis.

Differentially expressed genes in intestinal metaplasia correlate with previous reports. We found an over-expression of the genes *TFF1* (Trefoil Factor 1) and *VIL1* (Villin 1), well recognized as intestinal metaplasia markers 43. Interestingly, among the genes that showed an over expression, we found genes recognized as gastrointestinal stem cell markers (Table S1). Concretely, the following gastrointestinal stem cell markers were found: *LGR5* (Leucine-rich repeat containing G protein-coupled Receptor 5, FCh = 3.19), *PROM1* (Prominin 1, FCh = 2.91), and *OLFM4* (Olfactomedin 4, FCh = 4.24). Given these results, it is possible to infer that stem cells could play an important role in intestinal metaplasia. Additionally, mRNA metabolism is altered under this condition, in comparison with an early lesion, such as follicular gastritis.

Next, we aimed to determine whether there were also differences between chronic gastritis and intestinal metaplasia. Analysis of gene-set hallmarks displayed an enrichment of immunologically related genes in chronic gastritis, whereas in intestinal metaplasia, there was an enrichment of oxidative phosphorylation and several metabolism pathways (Table 5).

Ontological analysis showed an enrichment of immunological terms in chronic gastritis, indicating that an exacerbated immune response is increased in comparison with the immunological response in intestinal metaplasia. The results suggest that the inflammatory response plays an important role during chronic gastritis, which is absent in the intestinal metaplasia. On the other hand, mitochondrial-related terms were enriched in intestinal metaplasia, indicating that there was a down-regulation of these terms in chronic gastritis (Dataset S5). This is a trend similar to that previously observed when comparing chronic gastritis with follicular gastritis. This confirms the overall findings of a mitochondrial alteration in chronic gastritis, which is restored during progression into intestinal metaplasia. Pathway analysis revealed an enrichment of the metabolism of lipids, as well as the metabolism of xenobiotics and amino acids in intestinal metaplasia. In contrast, there was an enrichment of immunologic signaling pathways and proliferation pathways in chronic gastritis (Dataset S5).

Interestingly, expression analysis between these gastric entities resulted in only 31 genes with a differential expression among them (Dataset S6.). Of these, 16 genes displayed an overexpression, whereas, in intestinal metaplasia, 15 genes showed a down-regulation in expression with respect to chronic gastritis (Figure S1C). Despite the reduced number of genes, we performed a hierarchical clustering that demonstrated an expression pattern specific for each lesion (Figure S2C). Ontological analysis solely exhibited a non-significant enrichment in metabolism of nucleoside monophosphate. Similarly, pathway analysis only revealed enrichment in the sphingolipid metabolism (Table 3).

### Real time-PCR and immunohistochemistry analysis support the microarray findings

We performed real-time PCR of 10 genes of biological interest, which are associated with proliferation process, stem cell markers, transport of molecules, and mitochondria, which were differentially expressed in chronic gastritis and intestinal metaplasia. We selected genes that were up- and down-regulated and performed the assay in all the samples previously analyzed. We confirmed the up-regulation of *LRP1* (Low density lipoprotein receptor-related protein 1), *SLCO2A1* (Solute Carrier Organic Anion Transporter Family Member 2A1), and *MUC17* (Mucin 17) in chronic gastritis and of *PROM1* (Prominin 1), *OLFM4* (Olfactomedin 4), and *LGR5* (Leucine-rich repeat containing G protein-coupled Receptor 5) in intestinal metaplasia. The down-regulation of *NDUFS8* (NADH: Ubiquinone Oxidoreductase Core Subunit S8), *MT2A* (Metallothionein 2A), *HDAC7* = Histone Deacetylase 7, and *PDK1* (Pyruvate dehydrogenase kinase 1) in chronic gastritis and intestinal metaplasia, respectively, was also confirmed (Figure S3).

**Figure 1:**
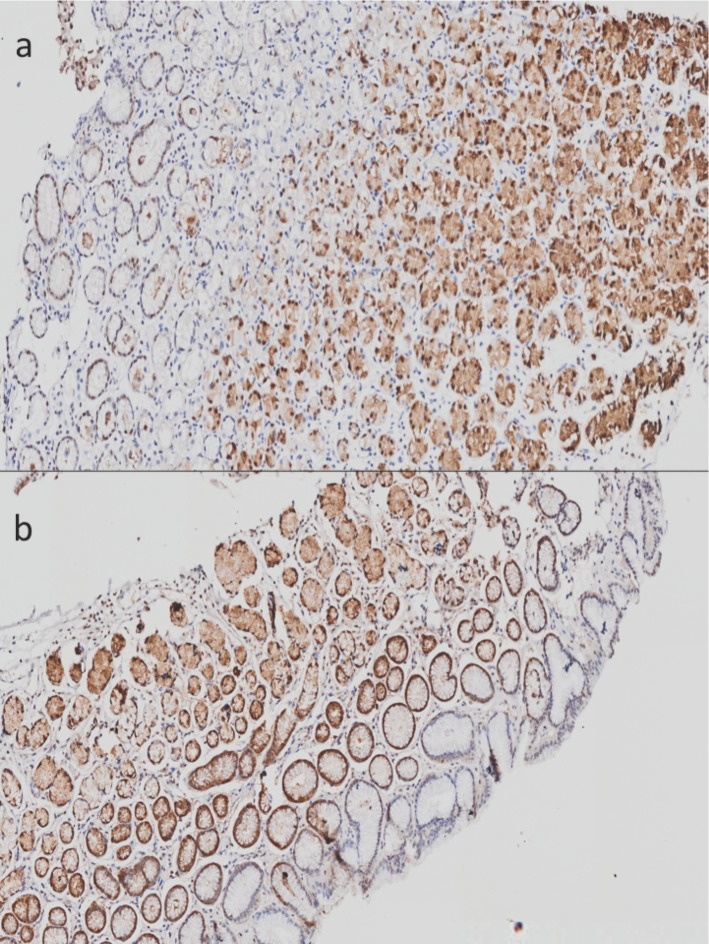
** Imunohistochemistry of Metallothioneins (MTs) in stomach tissue without histological lesions.** A) Tissue corresponding to gastric corpus without lesions. B) Tissue corresponding to gastric antrum without lesions. 20X amplification.

In addition to mitochondrial dysfunction, our attention was drawn to the down-regulation of members of the metallothionein family in chronic gastritis in comparison with follicular gastritis. We found a down-regulation of these molecules in chronic gastritis in comparison with follicular gastritis, despite the fact that both are inflammatory lesions. Given this, we sought the protein expression of metallothionein 1 and 2 in histopathological lesions of stomach, as well as in tissue without lesions. We performed a qualitative analysis of these proteins in order to determine their distribution within the cell and along the gastric tissue, which was not previously performed. Although the differences we found were between chronic and follicular gastritis, we also determined their expression in tissue without histological lesions. In these tissues we detected a positive signal for MTs, mainly in the glandular area of gastric tissue, despite the localization (antrum or corpus) (Figure 1). The signal was found mostly in the nucleus and, in a minor proportion, in the cytoplasm.

**Figure 2:**
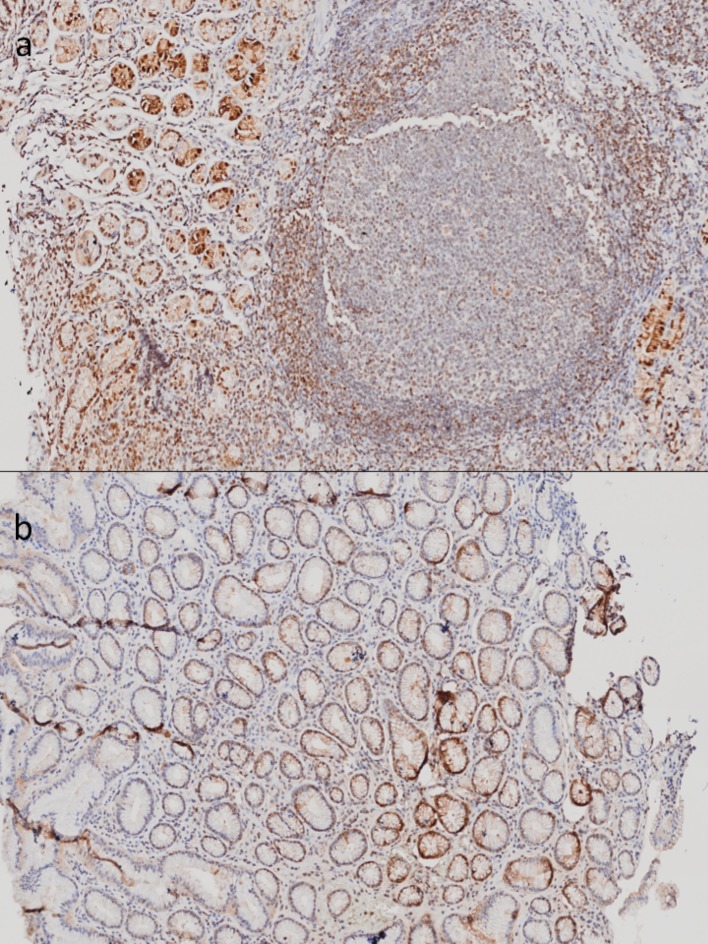
** Immunohistochemistry of Metallothioneins (MTs) of tissue from stomachs with follicular gastritis and chronic gastritis.** A) Samples corresponding to tissue with follicular gastritis, with the presence of well defined follicles. B) Tissue corresponding to chronic gastritis: the inflammatory infiltrate does not form structure follicles. 20X amplification.

In tissues corresponding to gastritis, we found an evident positive signal in epithelial and inflammatory cells (Figure 2). Even though we could not perform a quantitative analysis, we were able to identify that the stronger signal shown in follicular gastritis was due to the presence of lymphoid follicles (Figure 2A), which were absent in chronic gastritis (Figure 2B). This indicates that the differences observed in the microarray analysis could be due to the presence of these specific structures and could play a role during the occurrence of this lesion.

## Discussion

The objective of the present study was to determine the gene-expression profile of chronic gastritis, follicular gastritis and intestinal metaplasia lesions associated with the development of gastric adenocarcinoma. The information obtained improves the knowledge of the biological processes altered in each lesion.

It has been well established that *H. pylori* causes gastritis in infected subjects. In Mexico, infection rates reach 80% of the population over 20 years of age 44, resulting in a high incidence of gastritis 45. For that reason, we were unable to collect gastric biopsies of healthy stomachs for the gene-expression analysis. Nevertheless, we compared gene-expression patterns between gastric lesions and identified specific expression patterns distinguishing each lesion. The newest microarray analysis and new bioinformatic approaches have the advantage of generating multiple comparisons among study groups. These results provide a new perspective on the mechanisms that could be involved in the progression from gastritis to adenocarcinoma.

We were able to discern the different gene-expression patterns between follicular gastritis and chronic gastritis using different molecular approaches. This emphasizes the potential of molecular tools, such as microarrays or even RNA-sequencing (RNA-seq) in the analysis and molecular classification of histopathological lesions.

Lymphoid follicles are typically absent in normal gastric tissue, and in some cases, arise as a response to *H. pylori* infection, resulting in the development of follicular gastritis. This feature distinguishes the latter from chronic gastritis, in which the inflammatory reaction does not form a definite structure. When we compared the two variants of gastritis, we found a differential expression of mitochondrial-related genes, which were down-regulated in chronic gastritis in comparison with follicular gastritis. In particular, we found a down-regulation of genes involved in oxidative phosphorylation. Although these do not encode for enzymes involved in electron transport, they form part of the structure necessary for the good functioning of this pathway. An alteration in mitochondria, and particularly in oxidative phosphorylation, was also found in chronic gastritis using different bioinformatic approaches. This leads us to infer that mitochondrial disruption is, in fact, a characteristic feature of chronic gastritis.

The mitochondrion is an organelle that plays an important role in a great number of metabolic processes 46, such as oxidative phosphorylation, the main source of Reactive Oxygen Species (ROS) within the cell 47. It is suggested that an impairment in mitochondria electron transport is associated with an increase in the production of the free radicals involved in cell damage 48.

In oxidative phosphorylation, many proteins are required for the proper assembly of respiratory complexes 49. It is necessary to investigate whether the gene down-regulation found in this study in fact produces the impairment in mitochondria with a subsequent increase in the production of ROS.

It has been suggested that *H. pylori* can induce mitochondrial DNA (mtDNA) mutations and impair mitochondrial function, which might modulate gastric carcinogenesis 50. Our results suggest that mitochondria, and more specifically, oxidative phosphorylation, may play a crucial role in the development of chronic gastritis. Nevertheless, whether this feature is a cause, or a consequence of the inflammatory microenvironment remains to be determined.

The alteration of metallothioneins in stomach tissue has been identified previously. Boussioutas *et al.* 14 reported an alteration in the expression of some members of this family in gastritis, suggesting that the differential expression could be a result of *H. pylori* infection. In our search we were able to evaluate the protein expression of metallothioneins and identified a strong signal in chronic gastritis tissue. In particular, the stronger signal was found in tissue corresponding to follicular gastritis. The signal was observed not only in epithelial cells but also within follicle structure, suggesting that metallothioneins could have an interaction within immune cells. Although we could not perform a quantitative analysis of metallothionein expression, we were able to describe the expression pattern of these proteins within the gastric tissue. We found interesting differences in tissues with different types of gastritis, which to our knowledge, has not been described to date. It is noteworthy that this work included gastric tissue without histological lesions for immunohistochemistry of metallothioneins analysis.

Metallothioneins have been described as important players in cellular homeostasis. They are known as antioxidants that sense and inactivate ROS. They are responsive to interleukin stimulus and also function as anti-inflammatory molecules that protect against free radicals 51. Several animal models have revealed that metallothioneins play a crucial role in *H. pylori* infection. It has been demonstrated that deficiency in these molecules increases the inflammatory response in the stomach and the risk of the development of gastritis in animals 52-54. It has also been suggested that a lack of these molecules increases susceptibility to the development of carcinogenic lesions 52. Studies in human samples have described that subjects with gastric cancer infected with *H. pylori* had a lower expression of metallothioneins in comparison with infected subjects who did not have cancer. This suggests that their anti-carcinogenic role is also important in humans 53.

On the other hand, in follicular gastritis, we identified the enrichment of the glutamate-signaling pathway, and particularly in genes related to the glutamate receptor. Interestingly, this enrichment was found when comparing follicular gastritis with either chronic gastritis or intestinal metaplasia, indicating that this is a specific characteristic of follicular gastritis.

Previous studies have shown that the *H. pylori* virulence factor Gamma-Glutamyl Transpeptidase (GGT) has the capacity to convert glutathione and glutamine into glutamate, which is incorporated into the bacterial metabolism 55. However, glutamate also plays a role in immune cells, specifically, in dendritic cells and in T lymphocytes, which can recognize glutamate and induce a tolerogenic immune response against *H. pylori* infection 40. These results indicate that *H. pylori* could interact in several ways with the cells present in the gastric mucosa. Nevertheless, more functional studies are required to confirm the role of glutamate signaling in follicular gastritis.

On analyzing intestinal metaplasia, we found an over-expression of genes such as *TFF1* and *VIL1*, characterized as markers of intestinal metaplasia 43. The detection of this over-expression corroborates the reliability of our results.

Intriguingly, we found an unexpected enrichment of ontological genes and pathways related to RNA metabolism, particularly those involved in alternative splicing. This process is recognized as a crucial step in gene expression, and several studies have reported that a dysfunction in splicing could be related to the development of cancer 56, 57. It has been suggested that an incorrect assembly of the spliceosome, deregulated alternative splicing, or the accumulation of un-spliced mRNA could lead to an alteration of gene expression, as observed in leukemia and myelodysplasia 58. Given this information, alternative splicing could play an important role in intestinal metaplasia, regulating the expression of intestinal-associated genes or participating in the development of adenocarcinoma. The biological implications of these findings, however, remain to be deciphered.

In the gastrointestinal tract, it has been established that stem cells are responsible for maintaining the epithelial structure. Their role in the stomach has gained attention recently, and although the characteristics of gastrointestinal stem cells have not been fully elucidated, several related markers have been described 59, 60. In intestinal metaplasia, we found a surprising up-regulation of genes that encoded stem cell markers. Specifically, we found an increase in the expression of *PROM1* and *LGR5*. In addition, there was an important up-regulation in the expression of *OLFM4*. Although we did not find a statistical significance, this caught our attention due to its relationship with the other markers mentioned previously.

The cancer stem cell theory indicates that stem cells could be those that originate and maintain tumors 61. This has also been proposed for gastric adenocarcinoma, and various studies have investigated the hypothesis 62. A link between *H. pylori* and stem cells has also been reported. Using animal models, it has been shown that *H. pylori* is capable of invading progenitor cells through the glycan receptors present in stem cells 63. In addition, it has been reported that *H. pylori* can colonize the gastric glands where the stem cells reside and can alter their properties 64. Our results demonstrated a differential expression between intestinal metaplasia and precursor lesions such as gastritis. The up-regulation of stem cells in metaplasia of the stomach and the esophagus has been studied previously, suggesting that stem cells have an important role during intestinal metaplasia. A question remains concerning whether stem cells are involved in tissue transdifferentiation or participate in the progression to gastric adenocarcinoma. The study of these molecules will improve our understanding of their role in the origin and progression of intestinal metaplasia and gastric adenocarcinoma 62.

The microarray analysis was performed with a small number of samples. Nonetheless, the information obtained from this analysis was subjected to further validation using real-time PCR. Both techniques revealed consistent data that confirms the results presented.

In summary, this study provides a comprehensive gene-expression analysis of follicular gastritis, chronic gastritis, and intestinal metaplasia. We identify specific biological processes associated with the histological lesions analyzed. In lesions corresponding to gastritis, we found a signature that could be potentially associated with *H. pylori* infection. These results suggest that the bacterium has developed several mechanisms to interact with the gastric mucosa, which eventually could lead to the development of atrophy or intestinal metaplasia. In addition, the results observed in intestinal metaplasia could be associated with further progression to malignant lesions (S6 Dataset, Fig. S4). The results also open the door to more detailed studies of the relationship between lymphoid follicles and MALT. An approach such as that employed in this study could help to expand the figure presented and the association between *H. pylori* and gastric malignancies.

Microarray analysis has the advantage of generating multiple comparisons between study groups, and although the transcriptomic approach provided very interesting information, several molecular mechanisms in gastric lesions remain poorly understood. The potential of the newest high-throughput technologies and the new bioinformatic tools have allowed us to evaluate the whole-genome expression changes rather than just focusing on a limited number of genes. This provides a better view of biological processes, such as chronic gastritis, follicular gastritis and intestinal metaplasia involved in the development of gastric cancer. Furthermore, the results obtained in this study contribute a better understanding of the biological processes of these diseases based on the comparison of the characteristics of the gene-expression profiles in a conjugated manner.

## Supplementary Material

Supplementary figures and tables.

Dataset S1-S5.

Dataset S6.

## Figures and Tables

**Table 1:** Demographic characteristics of the study population

Samples corresponding to gastric biopsies obtained from endoscopy			
Group	Gender		Age (years) (Interval)
	M	F	
Follicular gastritis	2	5	48.4 (38-65)
Chronic gastritis	4	3	42.1 (38-49)
Intestinal metaplasia	2	5	61.4 (37-78)
Samples corresponding to FFPE tissue			
**Group**	**Gender**		**Age (years)** **(Interval)**
	**M**	**F**	
Gastric mucosa without alterations	5	13	49.1 (22-74)
Follicular gastritis	6	14	56.3 (31-95)
Chronic gastritis	8	13	63 (31-92)
Intestinal metaplasia	9	11	67.2 (43-82)

FFPE: Formalin-Fixed Paraffin-Embedded.

**Table 2:** Gene-set hallmarks enriched in the comparison of chronic gastritis *vs.* follicular gastritis obtained by GSEA

Follicular gastritis				
NAME	SIZE	NES	NOM p-val	FDR q-val
HALLMARK_KRAS_SIGNALING_DN	189	-1.4338	0.0079	0.5315
HALLMARK_OXIDATIVE_PHOSPHORYLATION	182	-1.3375	0.2230	0.5238
HALLMARK_MYOGENESIS	193	-1.2289	0.1780	0.6475
HALLMARK_HYPOXIA	199	-0.8808	0.7500	1
HALLMARK_ADIPOGENESIS	190	-0.8651	0.6338	1
HALLMARK_REACTIVE_OXYGEN_SPECIES_PATHWAY	48	-0.8473	0.6272	1
HALLMARK_BILE_ACID_METABOLISM	112	-0.7314	0.9626	1
HALLMARK_XENOBIOTIC_METABOLISM	187	-0.7276	0.9578	1
HALLMARK_ANDROGEN_RESPONSE	97	-0.7051	0.8198	1
HALLMARK_FATTY_ACID_METABOLISM	146	-0.6289	0.9144	0.9914
**Chronic gastritis**				
NAME	SIZE	NES	NOM p-val	FDR q-val
HALLMARK_TGF_BETA_SIGNALING	54	0.5849	0.0161	0.5285
HALLMARK_MITOTIC_SPINDLE	197	0.5467	0.0523	0.7653
HALLMARK_PROTEIN_SECRETION	96	0.5078	0.1930	1
HALLMARK_APOPTOSIS	161	0.4011	0.1567	1
HALLMARK_UV_RESPONSE_DN	140	0.4363	0.0692	0.8259
HALLMARK_INTERFERON_ALPHA_RESPONSE	97	0.4974	0.3308	1
HALLMARK_G2M_CHECKPOINT	191	0.5463	0.3866	1
HALLMARK_ESTROGEN_RESPONSE_EARLY	189	0.3268	0.1525	1
HALLMARK_E2F_TARGETS	181	0.5619	0.4048	1
HALLMARK_APICAL_JUNCTION	200	0.2944	0.2749	0.9846

GSEA: Gene Set Enrichment Analysis Size: Number of genes corresponding to the gene set NES: Normalized Enriched Score NOM p-val: Nominal p-value FDR q-val: False Discovery Rate q-value.

**Table 3:** KEGG pathways enriched in the comparisons

Chronic gastritis *vs.* follicular gastritis									
Term	Count			%		p-value	Fold Enrichment		FDR
hsa05016:Huntington disease	13			4.1009		2.55E-05	4.4247		2.83E-02
hsa05012:Parkinson disease	10			3.1546		1.90E-04	4.7863		2.11E-01
hsa05010:Alzheimer disease	11			3.4700		2.54E-04	4.1345		2.81E-01
hsa00190:Oxidative phosphorylation	9			2.8391		1.07E-03	4.2414		1.18E+00
h_etcPathway:Electron Transport Reaction in Mitochondria	2			0.6309		8.91E-02	20.5286		5.95E+01
hsa03320:PPAR signaling pathway	4			1.2618		9.94E-02	3.5516		6.87E+01
**Intestinal metaplasia *vs.* follicular gastritis**									
hsa00500:Starch and sucrose metabolism		5		1.1062	0.0232			4.5176	24.0029
hsa00511:Other glycan degradation		3		0.6637	0.0641			7.1152	53.8902
hsa00053:Ascorbate and aldarate metabolism		3		0.6637	0.0715			6.6967	57.9410
hsa00982:Drug metabolism		5		1.1062	0.0780			3.0603	61.2824
**Intestinal metaplasia *vs.* Chronic gastritis**									
hsa00600:Sphingolipid metabolism		2	7.4074		0.0377			43.4615	25.4056
									

KEGG: Kyoto Encyclopedia of Genes and Genomes.

**Table 4:** Gene-set hallmarks enriched in the comparison of intestinal metaplasia *vs.* follicular gastritis obtained by GSEA

Follicular gastritis				
NAME	SIZE	NES	NOM p-val	FDR q-val
HALLMARK_KRAS_SIGNALING_DN	189	-1.2434	0.1213	1
HALLMARK_INTERFERON_GAMMA_RESPONSE	188	-1.1809	0.3489	1
HALLMARK_INTERFERON_ALPHA_RESPONSE	97	-1.1693	0.3752	1
HALLMARK_IL6_JAK_STAT3_SIGNALING	85	-1.1606	0.3131	0.8984
HALLMARK_TNFA_SIGNALING_VIA_NFKB	194	-1.1438	0.3373	0.7671
HALLMARK_INFLAMMATORY_RESPONSE	199	-1.1387	0.3339	0.6487
HALLMARK_MYOGENESIS	193	-1.0408	0.4157	0.7506
HALLMARK_ALLOGRAFT_REJECTION	189	-0.9863	0.5048	0.7661
HALLMARK_IL2_STAT5_SIGNALING	186	-0.9640	0.5050	0.7222
HALLMARK_APICAL_SURFACE	44	-0.9447	0.5909	0.6846
**Intestinal metaplasia**				
NAME	SIZE	NES	NOM p-val	FDR q-val
HALLMARK_PROTEIN_SECRETION	96	1.4032	0.1712	1
HALLMARK_PEROXISOME	97	1.3501	0.0663	1
HALLMARK_E2F_TARGETS	181	1.3306	0.2116	1
HALLMARK_G2M_CHECKPOINT	191	1.3166	0.2317	1
HALLMARK_ESTROGEN_RESPONSE_EARLY	189	1.2874	0.1774	0.9926
HALLMARK_MYC_TARGETS_V1	183	1.2823	0.2949	0.8493
HALLMARK_PANCREAS_BETA_CELLS	38	1.2765	0.2205	0.7448
HALLMARK_ESTROGEN_RESPONSE_LATE	191	1.2593	0.1859	0.7066
HALLMARK_FATTY_ACID_METABOLISM	146	1.2525	0.2115	0.6472
HALLMARK_UV_RESPONSE_DN	140	1.2270	0.2145	0.6496

GSEA: Gene Set Enrichment Analysis Size: Number of genes corresponding to the gene set NES: Normalized Enriched Score NOM p-val: Nominal p-value FDR q-val: False Discovery Rate q-value

**Table 5:** Gene-set hallmarks enriched in the comparison between intestinal metaplasia *vs.* chronic gastritis obtained by GSEA

Chronic gastritis				
NAME	SIZE	NES	NOM p-val	FDR q-val
HALLMARK_TGF_BETA_SIGNALING	54	1.6599	0.0196	0.1973
HALLMARK_INTERFERON_ALPHA_RESPONSE	97	1.5760	0.0147	0.2244
HALLMARK_INTERFERON_GAMMA_RESPONSE	188	1.5739	0.0229	0.1536
HALLMARK_TNFA_SIGNALING_VIA_NFKB	194	1.5618	0.0324	0.1306
HALLMARK_COMPLEMENT	190	1.4035	0.1067	0.3856
HALLMARK_INFLAMMATORY_RESPONSE	199	1.3848	0.1097	0.3627
HALLMARK_IL2_STAT5_SIGNALING	186	1.3768	0.1166	0.3265
HALLMARK_APOPTOSIS	161	1.3517	0.1463	0.3330
HALLMARK_ALLOGRAFT_REJECTION	189	1.3498	0.1583	0.3005
HALLMARK_APICAL_JUNCTION	200	1.3454	0.1133	0.2771
**Intestinal metaplasia**				
NAME	SIZE	NES	NOM p-val	FDR q-val
HALLMARK_OXIDATIVE_PHOSPHORYLATION	182	-1.6100	0.0634	0.1887
HALLMARK_FATTY_ACID_METABOLISM	146	-1.4598	0.0603	0.4260
HALLMARK_ADIPOGENESIS	190	-1.3665	0.1183	0.5491
HALLMARK_BILE_ACID_METABOLISM	112	-1.2779	0.1393	0.6824
HALLMARK_XENOBIOTIC_METABOLISM	187	-1.2281	0.1572	0.7188
HALLMARK_PANCREAS_BETA_CELLS	38	-1.2124	0.2461	0.6432
HALLMARK_KRAS_SIGNALING_DN	189	-1.1851	0.1954	0.6200
HALLMARK_MYOGENESIS	193	-1.1576	0.2349	0.6099
HALLMARK_PEROXISOME	97	-1.1574	0.2520	0.5431
HALLMARK_ESTROGEN_RESPONSE_LATE	191	-1.0858	0.3596	0.6494

GSEA: Gene Set Enrichment Analysis Size: Number of genes corresponding to gene set NES: Normalized Enriched Score NOM p-val: Nominal p-value FDR q-val: False Discovery Rate q-value
