# Supplementary material for: Translation of gastric disease progression at gene level expression
Source: J Cancer. 2020 Jan 1;11(2):520–32. doi: 10.7150/jca.29038 (PMC6930440; doi:10.7150/jca.29038)
Supplement: Supplementary file 1 — Supplementary figures and tables. [file jcav11p0520s1.pdf]

Supplementary Information

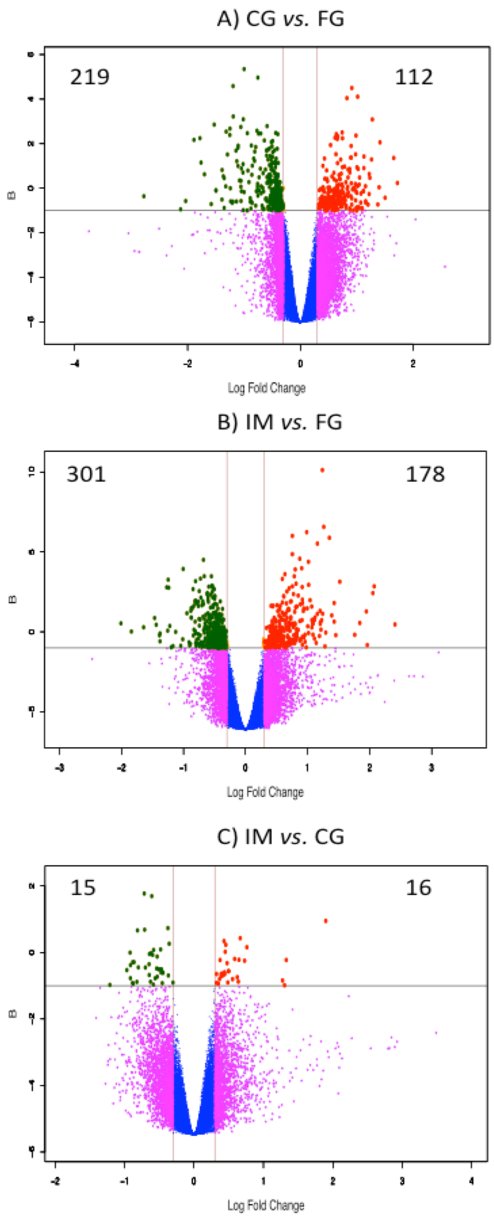

Figure S1. Volcano plots of differentially expressed genes. Volcano plots showing the differentially expressed genes: A) Volcano plot corresponding to comparison chronic gastritis *vs.* follicular gastritis, B) Volcano plot corresponding to comparison intestinal metaplasia *vs.* follicular gastritis, and C) Volcano plot corresponding to comparison intestinal metaplasia *vs.* chronic gastritis. Fold changes are represented in log2 base along the x-axis and the level of trust in the form of B-statistic ($B > -1$) along the y-axis. Red and green dots represent up and down-regulated genes respectively. The numbers in each quadrant indicate the number of genes selected. Other colors: magenta shows not statistically robust changes and blue shows low fold change with low statistical significance.

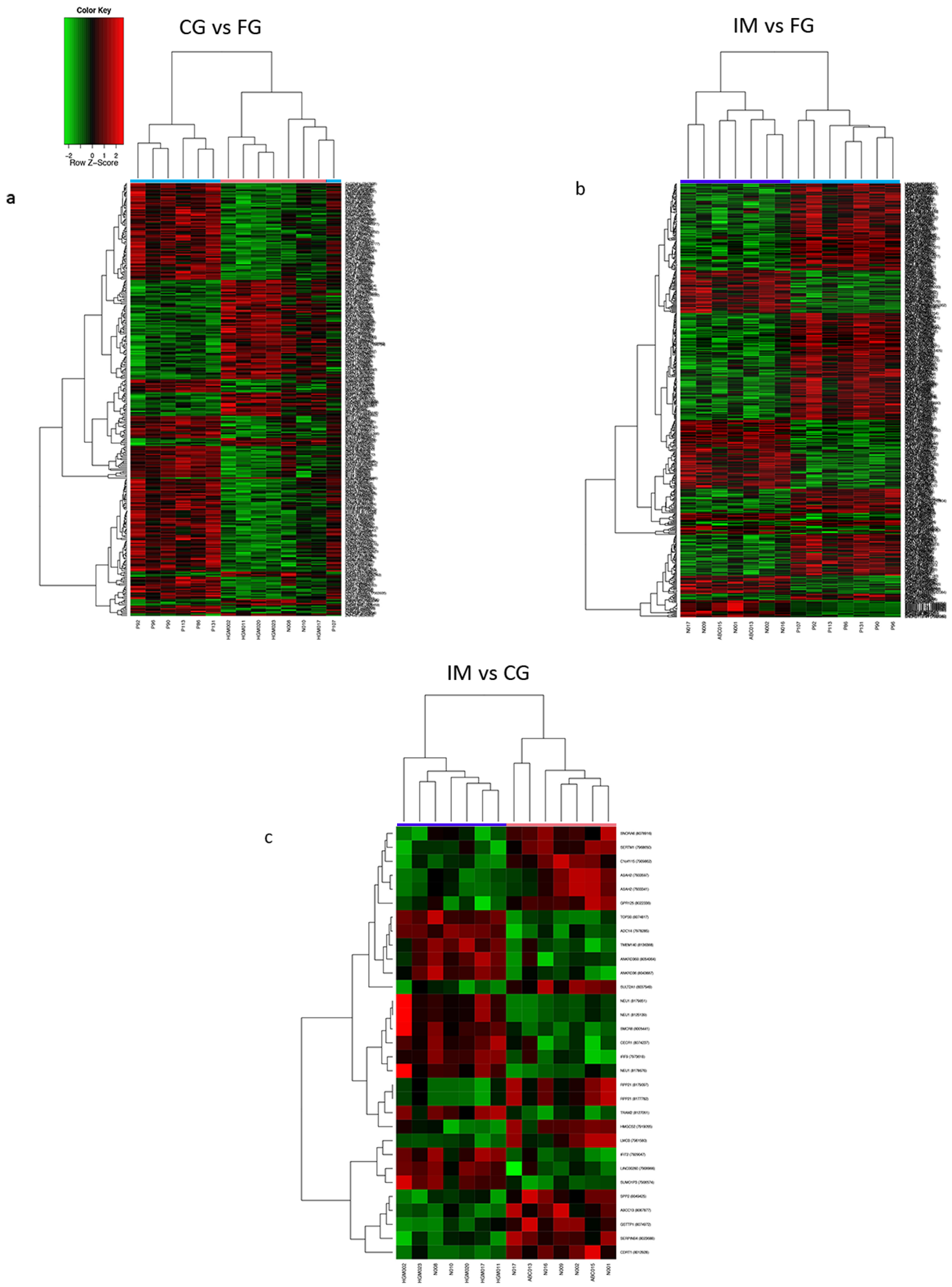

Figure S2. Hierarchichal clustering. Unsupervised clustering of differentially expressed genes. Each row represents individual differentially expressed genes, and columns represent each sample. The color red or green reflects up- or down-regulation, respectively. a) CG *vs.* FG, b) IM *vs.* FG, and c) IM *vs.* CG. The top bar in blue color represents the FG samples; top bar in pink CG samples and top bar in purple IM samples.

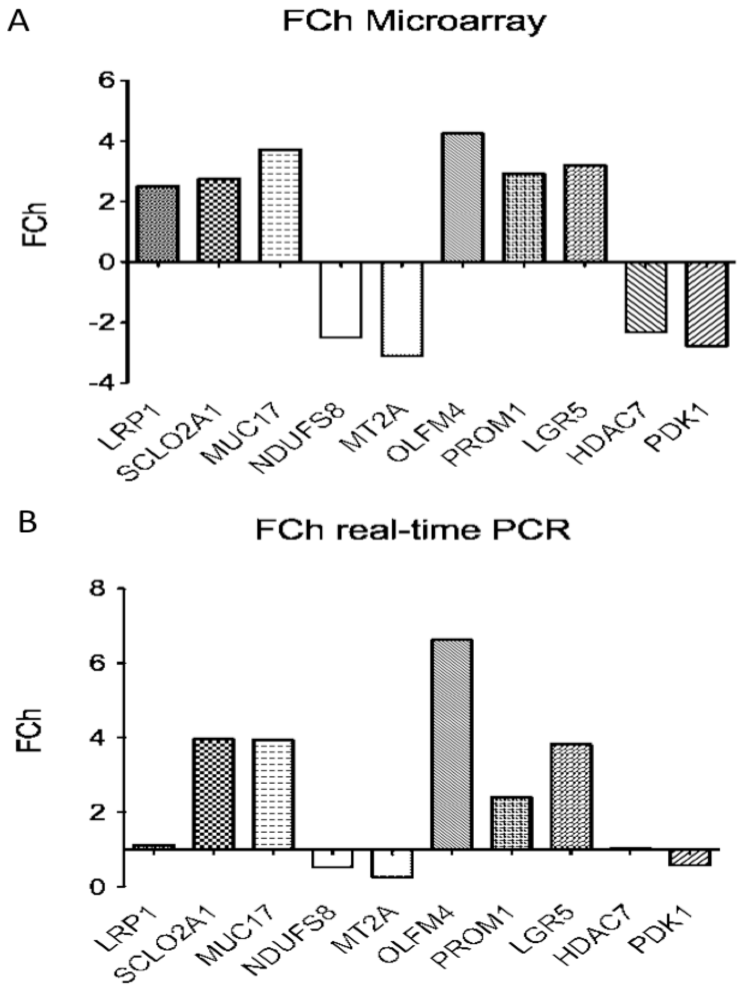

Figure S3. Real time PCR and microarray fold changes of genes validated. Fold change of genes of interest by A) microarray and B) real-time PCR. *LRP1* = Low density lipoprotein receptor-related protein 1; *SLCO2A1* = Solute Carrier Organic Anion Transporter Family Member 2A1; *MUC17* = Mucin 17; *NDUFS8* = NADH: Ubiquinone Oxidoreductase Core Subunit S8; *MT2A* = Metallothionein 2A; *OLFM4* = Olfactomedin 4; *PROM1* = Prominin 1; *LGR5* = Leucine-rich repeat containing G protein-coupled Receptor 5; *HDAC7* = Histone Deacetylase 7; *PDK1* = Pyruvate dehydrogenase kinase 1.

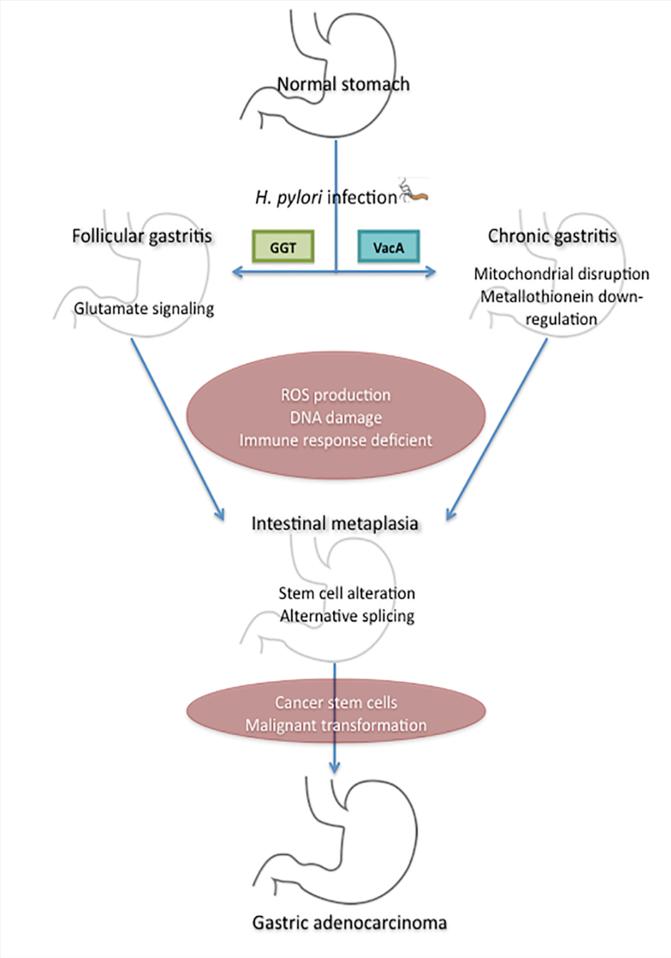

Figure S4. Summary of gene expression findings. Key expression findings are schematized for each lesion analyzed. The possible role of *H. pylori* and the development of gastric adenocarcinoma are represented.

Table S1. Demographic characteristics of study population. Samples corresponding to gastric biopsies obtained from endoscopy.

Clinical Entity	Gender	Age	Pathological Diagnosis
Follicular gastritis	M	38	Chronic follicular gastritis with activity associated to *H. pylori*, scarce amount of bacilli in the body and antrum
	M	44	Chronic follicular gastritis with moderated activity associated to *H. pylori* in the body and antrum
	F	43	Chronic follicular gastritis with moderated activity associated to *H. pylori* in the antrum
	F	46	Chronic follicular gastritis with moderate activity associated to *H. pylori*, moderate amount of bacilli in the antrum
	F	49	Chronic follicular gastritis with mild activity associated to *H. pylori*, scarce amount of bacilli in the body and antrum
	F	54	Chronic follicular gastritis moderate with moderate activity associated to *H. pylori* in the antrum
	F	65	Chronic follicular gastritis with moderate activity associated to *H. pylori*, scarce amount of bacilli in the body and antrum
Chronic gastritis	M	39	Moderate chronic gastritis
	M	43	Superficial chronic gastritis
	M	43	Moderate chronic gastritis
	M	49	Moderate chronic gastritis
	F	38	Mild superficial chronic gastritis
	F	39	Mild superficial chronic gastritis
	F	44	Moderate chronic gastritis
Intestinal metaplasia	M	ND	Complete intestinal metaplasia
	M	78	Intestinal metaplasia
	F	ND	Complete intestinal metaplasia
	F	37	Complete intestinal metaplasia with chronic atrophic gastritis. Abundant amount of *H. pylori* in the antrum
	F	46	Incomplete intestinal metaplasia with chronic atrophic gastritis
	F	72	Complete intestinal metaplasia
	F	74	Complete intestinal metaplasia

F = Female. M = Male. *H. pylori = Helicobacter pylori.* ND = No data

Supplementary Data Set

Dataset S1. Enrichment GSEA CG *vs*. FG. Gene sets corresponding to gene ontology terms and pathways enriched in follicular gastritis and chronic gastritis. (XLSX)

Dataset S2. Chart Ontology CG *vs*. FG. Ontology terms enriched in the differentially expressed genes between chronic gastritis and follicular gastritis. (XLSX)

Dataset S3. Enrichment GSEA IM *vs*. FG. Gene sets corresponding to gene ontology terms and pathways enriched in intestinal metaplasia and follicular gastritis. (XLSX)

Dataset S4. Chart Ontology IM *vs*. FG. Ontology terms enriched in the differentially expressed genes between intestinal metaplasia and follicular gastritis. (XLSX)

Dataset S5. Enrichment GSEA IM *vs*. CG. Gene sets corresponding to gene ontology terms and pathways enriched in intestinal metaplasia and chronic gastritis. (XLSX)

Dataset S6. Differentially expressed genes. List of the differentially expressed genes find on each of the contrast evaluated
